# Supplementary material for: Out of thin air: surveying tropical bat roosts through air sampling of eDNA
Source: PeerJ. 2023 Apr 26;11:e14772. doi: 10.7717/peerj.14772 (PMC10148639; doi:10.7717/peerj.14772)
Supplement: Supplemental Information 1 [file peerj-11-14772-s001.pdf]

Species	Sampler A		Sampler B		Sampler C		Sampler D		Sampler E		Sampler F	
Carollia perspicillata	4548	0	0	19	10	8198	43	7963	0	2462	0	0
Glossophaga mutica	25847	32	95389	26797	58	0	6508	75225	8	16713	21574	9989
Natalus mexicanus	0	0	3672	82134	2	11200	17	1177	66429	7241	28605	10556
Saccopteryx bilineata	12	0	0	0	0	38	0	0	0	0	0	0
Sturnira parvidens	0	29	0	2265	0	383	0	6208	0	0	19860	0
Trachops cirrhosus	14	0	1964	0	0	0	14142	0	0	31908	0	0
Alouatta palliata	0	0	0	20390	0	0	0	0	0	0	0	0
Bos taurus	35	144	0	16	51	78	0	2	30865	58	41075	0
Canis sp.	13164	311	3149	699	65	104327	0	3006	15	2395	13	5625
Equus caballus	0	0	0	0	0	0	4100	0	0	0	0	0
Leptodactylus fragilis	0	0	13	0	0	187	0	0	0	5255	0	0
Ototylomys phyllotis	0	0	0	0	0	0	5	0	13282	0	23	0
Ovis aries	36565	159	8254	0	127	0	49444	0	6	0	9	0
Scinax staufferi	0	0	0	0	0	0	0	0	1516	0	0	0
Sus scrofa	55170	14	0	0	0	112	0	0	0	36	0	0
Sylvilagus floridanus	8820	0	0	0	0	0	0	0	0	0	0	0
Trachycephalus typhonius	0	0	3952	0	0	0	0	0	0	0	0	0

Day Night

Figure S1. The total read count by sampler (summed across 3 PCR replicates) for each species detected during the day (approximately 8:30-15:00 - yellow) and at night (approximately 15:00-8:30 - blue) in the Schoolhouse Cave on April 28th- 29th, 2022.
