# Supplementary material for: Out of thin air: surveying tropical bat roosts through air sampling of eDNA
Source: PeerJ. 2023 Apr 26;11:e14772. doi: 10.7717/peerj.14772 (PMC10148639; doi:10.7717/peerj.14772)
Supplement: Supplemental Information 2 [file peerj-11-14772-s002.pdf]

A)

Species	Sampler A						Sampler B						Sampler C					
	R1	*R2*	*R3*	*R1*	*R2*	*R3*	*R1*	*R2*	*R3*	*R1*	*R2*	*R3*	*R1*	*R2*	*R3*	*R1*	*R2*	*R3*
Carollia perspicillata	4544	4	0	0	0	0	0	0	0	7	7	5	0	10	0	8193	5	0
Glossophaga mutica	25810	22	15	0	32	0	15082	51501	28806	33	10693	16071	26	32	0	0	0	0
Natalus mexicanus	0	0	0	0	0	0	0	3672	0	24381	39784	17969	0	2	0	705	10495	0
Saccopteryx bilineata	2	0	10	0	0	0	0	0	0	0	0	0	0	0	0	24	0	14
Sturnira parvidens	0	0	0	29	0	0	0	0	0	2265	0	0	0	0	0	0	383	0
Trachops cirrhosus	0	9	5	0	0	0	0	1964	0	0	0	0	0	0	0	0	0	0
Alouatta palliata	0	0	0	0	0	0	0	0	0	20390	0	0	0	0	0	0	0	0
Bos taurus	0	16	19	47	97	0	0	0	0	16	0	0	22	29	0	66	0	12
Canis sp.	0	0	13164	72	239	0	0	0	3149	699	0	0	26	39	0	41744	8233	54350
Equus caballus	0	0	0	0	0	0	0	0	0	0	0	0	0	0	0	0	0	0
Leptodactylus fragilis	0	0	0	0	0	0	0	0	13	0	0	0	0	0	0	0	187	0
Ototylomys phyllotis	0	0	0	0	0	0	0	0	0	0	0	0	0	0	0	0	0	0
Ovis aries	21229	6	15330	58	101	0	0	0	8254	0	0	0	55	72	0	0	0	0
Scinax staufferi	0	0	0	0	0	0	0	0	0	0	0	0	0	0	0	0	0	0
Sus scrofa	0	55170	0	0	14	0	0	0	0	0	0	0	0	0	0	0	112	0
Sylvilagus floridanus	0	0	8820	0	0	0	0	0	0	0	0	0	0	0	0	0	0	0
Trachycephalus typhonius	0	0	0	0	0	0	0	3952	0	0	0	0	0	0	0	0	0	0

Day Night

B)

Species	Sampler D						Sampler E						Sampler F					
	R1	*R2*	*R3*	*R1*	*R2*	*R3*	*R1*	*R2*	*R3*	*R1*	*R2*	*R3*	*R1*	*R2*	*R3*	*R1*	*R2*	*R3*
Carollia perspicillata	0	25	18	7938	17	8	0	0	0	0	0	2462	0	0	0	0	0	0
Glossophaga mutica	0	6	6502	29284	39742	6201	4	4	0	16696	8	9	8	21561	5	9936	31	22
Natalus mexicanus	8	9	0	1171	6	0	45759	20670	0	0	7241	0	47	28553	5	7054	3491	11
Saccopteryx bilineata	0	0	0	0	0	0	0	0	0	0	0	0	0	0	0	0	0	0
Sturnira parvidens	0	0	0	6208	0	0	0	0	0	0	0	0	19860	0	0	0	0	0
Trachops cirrhosus	14142	0	0	0	0	0	0	0	0	15446	16462	0	0	0	0	0	0	0
Alouatta palliata	0	0	0	0	0	0	0	0	0	0	0	0	0	0	0	0	0	0
Bos taurus	0	0	0	2	0	0	0	30846	19	43	0	15	0	15	41060	0	0	0
Canis sp.	0	0	0	0	0	3006	0	15	0	0	0	2395	0	13	0	0	0	5625
Equus caballus	0	0	4100	0	0	0	0	0	0	0	0	0	0	0	0	0	0	0
Leptodactylus fragilis	0	0	0	0	0	0	0	0	0	4181	0	1074	0	0	0	0	0	0
Ototylomys phyllotis	0	0	5	0	0	0	0	4	13278	0	0	0	14	9	0	0	0	0
Ovis aries	27461	21977	6	0	0	0	6	0	0	0	0	0	9	0	0	0	0	0
Scinax staufferi	0	0	0	0	0	0	1516	0	0	0	0	0	0	0	0	0	0	0
Sus scrofa	0	0	0	0	0	0	0	0	0	21	10	5	0	0	0	0	0	0
Sylvilagus floridanus	0	0	0	0	0	0	0	0	0	0	0	0	0	0	0	0	0	0
Trachycephalus typhonius	0	0	0	0	0	0	0	0	0	0	0	0	0	0	0	0	0	0

Day Night

Figure S2. The total read count by sampler (**A)** samplers A-C, **B)** samplers D-E) and PCR replicate (R#) for each species detected during the day (approximately 8:30-15:00 - yellow) and at night (approximately 15:00-8:30 - blue) in the Schoolhouse Cave on April 28th-29th, 2022.
